# Kerala’s progress towards universal health coverage: the road travelled and beyond

**DOI:** 10.1186/s12939-024-02231-2

**Published:** 2024-08-05

**Authors:** G.S. Adithyan, Alok Ranjan, V. R. Muraleedharan, T. Sundararaman

**Affiliations:** 1 Junior Public Health Professional (PHC), Department of UHC / Health Systems, WHO-SEARO, New Delhi, India; 2 Department of Humanities and Social Sciences, IIT Madras, Chennai, India; 3 School of Liberal Arts, Centre for Emerging Technology for Sustainable Development, Centre for Digital Health, IIT Jodhpur, Jodhpur, India; 4 Former Executive Director, NHSRC, New Delhi, India

**Keywords:** Equity, FHC, Kerala, PHC, UHC

## Abstract

**Background:**

Kerala has initiated many Universal Health Coverage (UHC) reforms in the last decade. The Aardram Mission launched in 2017 stands out owing to its scope, objectives, and commitments for strengthening Primary Health Care (PHC) in the State. The current study proposes to explore access and financial protection through the lens of equity in Kerala especially in the context of major UHC reforms carried out during the last decade. This paper will also highlight the key lessons from Kerala’s approach towards UHC and health systems strengthening through a political economy approach.

**Methods:**

Data from the Kerala state sample of 75th Round (2017-18) National Sample Survey is used for this study. Comparison is also drawn from the 71st Round Sample Survey, 2014, to measure the state’s progress in terms of access and financial protection. Logistic regression was used for the calculation. The findings were further explored through a political economy approach.

**Results:**

The share of public facilities for outpatient care is 47.5%, which is a significant increase from 34.0% (in 2014) in the state. The share of public sector for out-patient care has increased for the lower socio-economic population in the state. The share of public sector for in-patient care has also increased to 37.3% in 2017-18 from 33.9% in 2014, but not to the extent as the increase shown in outpatient care. The average out-of-pocket-expenditure during hospitalization has increased more in private facilities as compared to public for both outpatient care and hospitalization.

**Conclusions:**

Overall increase in the share of public facilities for both outpatient care and hospitalization is indicative of the enhanced trust among the people at large of the public healthcare delivery system in Kerala, post the launch of UHC reforms in the State. The insurance linked UHC reforms would be insufficient for the State to progress further towards UHC. Kerala with a long and successful history in ‘public provisioning’ should focus more on strengthening PHC through Aardram Mission in its journey towards pursuit of UHC.

**Supplementary Information:**

The online version contains supplementary material available at 10.1186/s12939-024-02231-2.

## Background

Kerala’s health sector performance has always gathered attention across the world for better health outcomes, on par with developed nations, even though Kerala’s per capita income is far below than them [1]. Even with the impressive health targets, the State is grappling with high prevalence of preventable and manageable noncommunicable diseases, emerging and re-emerging epidemics and outbreaks, and higher utilization of private sector, mostly un-regulated [2, 3]. Through the Universal Health Coverage (UHC) lens, this has negative implications on utilization of public facilities especially on primary care facilities as gate keepers and financial protection owing to high out of pocket expenses in private sector. This scenario is common in most of the Indian States, though in varied degrees [4].

Understanding the challenges ahead, Kerala pioneered many health systems strengthening reforms and UHC initiatives during the last decade. Among the major initiatives that was carried out during last decade, a notable one was the UHC Primary Healthcare (PHC) Pilot Project with support from University of East London which was conceived in 2012 [5]. The pilot project focused on expanding service coverage, strengthening existing primary care centers, and addressing the population level needs of the community. The pilot project sites selected were Community Healthcare Center, Venpakal and the Primary Healthcare Centers in Kallikad and Chemmaruthy in Trivandrum district. This initiative paved way to the introduction of the ‘Aardram Mission’ in 2017 on a pilot basis, which was later expanded progressively throughout the State with the prime objective of ‘re-engineering Primary Health Centres to Family Health Centres (FHC)’ [6]. This was one among the largest investment the State has made in PHC strengthening in terms of financial investment and the wider scope it envisaged [7]. The recent evaluation reports of Aardram Mission have shown mostly positive outcomes, in-terms of increased out-patient numbers, patient friendliness and improved service delivery [8].

In the publicly funded health insurance (PFHI) front, Kerala started with Comprehensive Health Insurance Scheme (CHIS) and Rashtriya Swasthya Bima Yojana (National Health Insurance) in late 2008, which later were modified and merged. According to one recent estimate, Kerala with just over 3 per cent of India’s population, accounted for over 40 per cent of the nationwide claims under the RSBY-CHIS [9]. Kerala also pioneered with many innovations in the PFHIs; two notable schemes include the CHIS-plus in 2011 which provided additional Rs 70,000 to RSBY-CHIS eligible households seeking care for chronic conditions relating to heart, kidney, liver, and trauma care and Karunya Benevolent Fund (KBF), in 2012 managed by the State Lotteries Department (Taxes) for providing financial assistance to acute ailments like Cancer, Haemophilia, Kidney and Heart diseases and for Palliative Care [10].

Later in 2018, when the Central Government of India launched the Ayushman Bharat – Pradhan Mantri Jan Arogya Yojana (PMJAY), after initial phase of disagreements, Kerala opted to bring together its various insurance and financial assistance schemes and launched an improved version of its own health insurance scheme, the Karunya Arogya Suraksha Padhathi (KASP). From July 2020, the scheme is being implemented directly by Government of Kerala under ‘trust mode’ through the newly constituted State Health Agency (SHA) [10]. Despite having a long history in implementing various PFHI schemes and higher share of budget allocation for health by the state compared to other states in India, Kerala has the highest Out of pocket expenditure (OOPE) on health [11–13].

Study done in recent years to understand the financial protection through equity lens under PFHIs in Kerala has shown the marginal effect. A cross sectional household survey conducted in four district of Kerala in 2019 has found that median OOPE for hospitalization under PFHIs was INR 9000 whereas without PFHIs it was INR 10,500 [14]. Various other studies in different states have also shown poor financial protection under PFHIs [15, 16]. In the given context, the current study proposes to explore access and financial protection through the lens of equity in Kerala, in the context of major UHC reforms carried out during the last decade through secondary data analysis of National Sample Survey (NSS) 75th Rounds (2017-18). Even though the findings from secondary data analysis of NSS 75th Rounds is not adequate to provide a comprehensive impact assessment of the specific reforms namely Aardram Mission or CHIS, this will provide indications and throw light relating to some broader implications of the UHC reforms happening in the State through a social lens.

This paper will also highlight the key lessons from Kerala’s approach towards UHC and health systems strengthening through a political economy approach. Health systems are complex and dynamic systems influenced by the historical contexts, power relations, values, culture, polities, governance, micro and macroeconomics etc. A ‘political economy of health’ approach includes how the production and consumption of healthcare services is influenced by markets and by state actions [17]. Hence, the paper will also briefly examine key lessons from the history and evolution of social-political reforms and movements in Kerala that will provide a critical reflection on factors within and outside health systems that have driven and shaped specific healthcare reforms.

## Methods

This study used Kerala state level data from the two consecutive rounds of National Sample Survey (NSS) on health which were conducted in 2014 (71st Round) and 2017-18 (75th Round) [4]. The surveys were conducted by the National Sample Survey Office, Ministry of Statistics and Program Implementation, Government of India and anonymized data was provided in the public domain for the research purposes. Since this study was based on the secondary data sets, there was no separate requirement of ethical clearance from the institutional review board.

Sample size of 71st NSS was 3,33,104 individuals at the All-India level, out of that 11,229 sample (rural: 5484, urban: 5745) were selected through multi-stage random sampling from the State of Kerala in 2014. On the other hand, 5,55,115 individuals were sampled at the All-India level in 75th NSS in 2017-18, and out of that sample 19,801 individuals were selected from Kerala (rural: 10,682; urban: 9,119). Sampling methodology remains the same in both the rounds of NSS. Similarly, interview schedules for the survey almost remains the same except few alterations in the 75th NSS.

NSS is self-reported survey and it collected socioeconomic information including place of residence, gender, social group, education, occupation, and consumption expenditure; and morbidity and mortality information. In the unit level data, place of residence was categorized as ‘rural’ and ‘urban’ areas and it was retained in the analyses. Gender was categorized as ‘male’, ‘female’, and ‘transgender’. However, there was no sample of transgender population in Kerala’s state sample. Social group, referred to caste and this was categorized as Scheduled Tribe (ST), Scheduled Caste (SC), Other Backward Class(OBC), and General (GEN). Education was taken in detail and for analysis it was categorized into four groups as ‘not literate’, ‘up to primary’, ‘up to secondary’, and ‘above secondary’. In unit level data ‘household type’ was asked based on the head of the household’s occupation, and though taken in more detail, for analyses purpose this study categorized it as ‘self-employed’, ‘regular wages’, and ‘casual labour’.

Households were asked about their usual monthly expenditure and it was used to calculate usual monthly per capita consumer expenditure (UMPCE). UMPCE was multiplied by 12 to calculate “usual annual” per capita consumer expenditure (UAPCE), and it was used in generating the quintiles for the rural and urban areas separately. Quintiles were categorized as poorest, poor, middle, rich, and richest. Quintiles were used as for categorizing the households and individuals under different economic strata.

Morbidity information was collected where individuals were asked if they fell sick in the last 15 days, and if so, detailed information including type of provider, medical and non-medical expenditure, and source of financing were asked. Similarly, individuals were also asked if they were hospitalized in the last 365 days, and if so, detailed information including medical and non-medical expenditure, type of healthcare facility, and reimbursement from the insurance company or employer were enquired. Apart from that NSS also collected data related to immunization, maternal health, and elderly care.

Individuals self-reported nature of ailment under 63 categories which were further categorized under 15 broad categories for this study as follows: infections, cancer, blood disease, endocrine and metabolic disease including diabetes, psychiatric and neurological, genitourinary, eye and ear, cardio-vascular including hypertension, respiratory, gastrointestinal, skin, musculoskeletal, injuries, obstetric, and unclassified conditions. Similar classification was also used in other studies [11].

Number of hospitalizations per 100 populations in the last 365 days was used for calculating the hospitalization rate. Similarly, number of individuals who reported suffering from the chronic ailment in the last one month, or any ailment in the last 15 days, or both, per 100 population was used in calculating the proportion of ailing population (PAP), which is a morbidity indicator.

71st NSS collected data about the healthcare provider during hospitalization and out-patient care. For analyses, all providers were categorized as public or private providers. Public providers included community health workers, health sub-centers, primary health centers, community health centers, district and sub-district hospitals, and government medical colleges. Private providers included private clinics and private hospitals. However, in the 75th NSS there was an additional subcategorization of non-governmental organization or trust hospital, and informal provider under the private sector. Informal providers are those who do not have legal permission for medical practice in India.

Health insurance coverage was also asked and this was categorized as government sponsored (example-Rastriya Swathya Bima Yojana), Schemes for government employees (example: Central Government Health Insurance Schemes), Employer supported health protection (example: Employees’ State Insurance Scheme), private voluntary insurance, others, and not covered. In this study, government sponsored insurance coverage is also referred to as Publicly Funded Health Insurance (PFHI).

Total medical (doctor’s consultation, medicine, diagnostics, bed charges, other medical expenditure), and non-medical (transportation, other expenditure such as food) expenditure was collected in NSS. Out-of-pocket expenditure (OOPE) was calculated after adding medical expenditure and transportation cost followed by deduction of any reimbursement provided. Certain cells of medical and non-medical expenditure were reported as missing value, because no expenditure was incurred or because it was included in package costs. These missing values were replaced as ‘0’ during analysis. Expenditure on childbirth was included in our OOPE calculation. There are various studies which have included [18–20] or excluded [21, 22] childbirth while estimating OOPE in India. Though childbirth is not an illness but a wellness event, it has been medicalized and commercialized, and it suffers from supplier induced demand [23]. However, inclusion or exclusion of childbirth from average OOPE calculation does not change the estimates significantly for Kerala [22]. The methodology for OOPE calculation we have used is similar to other earlier studies [20].

In conformity with earlier studies, if total OOPE during hospitalization was 10% or more to the UAPCE, it was categorized as catastrophic health expenditure at 10% threshold (CHE-10). Similarly, CHE-25, was calculated if total OOPE during hospitalization was 25% or more to the usual annual per capita expenditure [11–13].

Three logistics regression models were used to understand the factors determining hospitalization, PAP, and CHE-10. For model 1, incidence of hospitalization was the dependent variable whereas the socio-economic background characteristics: place of residence, gender, social groups, education category, occupation category, economic quintiles, and insurance coverage were the independent variables. Similarly, for model 2, reporting of ailment in the last 15 days or suffering from the chronic ailment was the dependent variable and independent variables remain the same like model 1 except insurance coverage. In the model 3, incidence of CHE-10 was the dependent variable whereas independent variables remain the same as in model 1, with addition of type of provider. Selection of independent variables was done based on literature review [18, 20, 24]. Variation inflation factors were checked and there was no multicollinearity among independent variables.

Analytical weight was applied for the descriptive statistics, which was provided by the NSS itself. All estimates, except logistic regression (Table 1), are after applying weightage to the sample. STATA 15 version was used for the analyses purpose.

## Results

Results of the study is presented under following sub-sections: (1) Hospitalization rate, PAP and Disease burden in Kerala, (2) Access to healthcare in Kerala, and (3) Financial protection in Kerala.

### Hospitalization rate, PAP and disease burden in Kerala

Total hospitalization rate in Kerala was 9.9% in 2017-18, which was significantly lower than 2014 (12.1%) (Additional File S1). Hospitalization rate was higher in rural (10.7%) areas compared to urban (8.9%) areas, male (10.5%) compared to female (9.3%), general category (9.6%) compared to ST (7.1%), and urban richest quintile (10.9%) compared to the rural poorest (9.9%) quintile in 2017-18. Reporting of overall PAP in Kerala has shown a downward trend from 30.8%, in 2014, to 24.5%, in 2017-18 (Additional File S1). PAP was higher in rural areas compared to urban areas, female compared to male, general category compared to ST category, and richest quintiles compared to poorest quintile. The insurance coverage also shows that there is higher coverage among the socially and economically disadvantaged sections, which is to be expected but not adequate as more than 50% of these categories are still not covered.

Logistic regression model shows odds of reporting hospitalization in the richest quintile was 1.65 times (95% CI: 1.45–1.88; *p* < 0.001) higher than the poorest quintile and it was statistically significant. Also, having PFHIs coverage increases the chance of hospitalization by 1.16 times compared to population which do not have any health insurance coverage. Similarly, population which had other types of insurance such as CGHS, ESIS, or private insurance had 1.46 times of hospitalization compared to population without health insurance coverage in Kerala (Table 1). Chances of reporting PAP in the elderly population (60 years or above) was 8.67 times (95% CI: 7.23–10.40; *p* < 0.001) higher than 0–4 years and it was statistically significant (Table 1).

**Table 1:** Factors affecting hospitalization, PAP, CHE-10 in Kerala in 2017-18 disaggregated by background characteristics

Total	Reporting of hospitalization (*N* = 19,801)	Reporting of PAP (*N* = 19,801)	CHE-10 (*N* = 3325)
**Age group (years, ref: 0–4 years)**			
5–14 years	0.93 (0.75–1.16)	0.69 (0.55–0.85)*	1.15 (0.53–2.48)
15–29 years	1.28 (1.2–1.59)*	0.57 (0.45–0.72)**	3.61 (1.81–7.18)**
30–44 years	1.43 (1.16–1.77)*	1.07 (0.86–1.32)	4.97 (2.52–9.82)**
45–59 years	2.46 (2.03–2.99)**	3.18 (2.62–3.85)**	4.84 (2.50–9.34)**
60 + years	4.48 (3.74–5.36)**		4.71 (2.50–8.88)**
**Place of Residence (ref: rural)**			
Urban	0.95 (0.88–1.03)	0.93 (0.86–1.01)	0.51 (0.42–0.62)**
**Gender (ref: male)**			
Female	0.85 (0.79–0.91)**	1.17 (1.08–1.26)**	0.77 (0.65–0.91)**
**Social Groups (ref: ST)**			
SC	1.22 (0.85–1.74)	1.41 (0.97–2.04)	1.69 (0.73–3.89)
OBC	1.20 (0.86–1.68)	1.41 (0.99-2.00)	1.70 (0.77–3.74)
General	1.11 (0.78–1.56)	1.28 (0.90–1.83)	1.99 (0.89–4.44)
**Education (ref: not literate)**			
Up to primary	0.73 (0.63–0.86)**	1.04 (0.88–1.22)	1.20 (0.83–1.74)
Up to secondary	0.57 (0.48–0.67)**	0.76 (0.64–0.90)*	1.08 (0.74–1.15)
Above Secondary	0.48 (0.39–0.57)**	0.57 (0.47–0.69)**	1.56 (1.02–2.37)
**Household occupation (ref: self-employed)**			
Regular Wages	1.01 (0.91–1.12)	0.87 (0.78–0.96)*	0.88 (0.71–1.10)
Casual Laborer	1.06 (0.96–1.17)	0.93 (0.85–1.03)	1.00 (0.81–1.24)
**Economic quintile (ref: poorest)**			
Poor	1.12 (1.01–1.25)*	1.33 (1.19–1.47)**	0.54 (0.39–0.76)
Middle	1.23 (1.09–1.38)**	1.64 (1.46–1.84)**	0.60 (0.44–0.82)*
Rich	1.51 (1.35–1.69)**	1.68 (1.49–1.88)**	0.46 (0.33–0.63)**
Richest	1.65 (1.45–1.88)**	2.28 (2.00-2.60)**	0.33 (0.24–0.45)**
**Insurance coverage (ref: No)**			
PFHI	1.16 (1.07–1.26)**	1.33 (1.22–1.45)**	0.72 (0.60–0.87 )*
Others (CGHS, private insurance, ESIS)	1.46 (1.27–1.68)**	1.20 (1.04–1.39)	0.47 (0.36–0.63) **
**Provider (ref: public)**			
Private	NA	NA	0.08 (0.03–0.22)**
**Model details**			
Number of observations	20,550	19,815	3279
LR Chi 2	1316.46	3972.83	807.84
Prob > Chi2	0.000	0.0000	0.000
Pseudo R2	0.067	0.1830	0.178
Log likelihood	-9169.99	-8866.38	-1859.29

Note: Given values are odds ratios and values in the parentheses and 95% confidence intervals; (**) *p*-value < 0.001, (*) *p*-value < 0.05; Source: Authors’ computation from unit records of NSSO 75th Round 2017-18

Infectious diseases were the major cause (28.5%) of hospitalization, followed by cardiovascular conditions (12.9%) and injuries (9.4%) in Kerala (Additional File S2). On the other hand, for outpatient care, cardiovascular conditions (27.1%) and diabetes (20.4%) were the major causes of Out-Patient (OP) visit in Kerala. The highest utilization of public sector for hospitalization episodes was for blood diseases and cancers and the lowest was for skin diseases. For out-patient care, Cancer (69.8%) and Cardiovascular diseases (51.1%) accounts for the maximum share treated under public sector and again skin diseases (23.6%) being the lowest.

### Access to healthcare

Access to healthcare is a broader term used in various context of service delivery. In this study, we have used utilization rate as a proxy indicator for the access to healthcare.

#### Hospitalization

Overall public healthcare utilization in Kerala increased from 33.9%, in 2014, to 37.3%, in 2017-18 (Table 2) for In-Patient (IP) care. Under private healthcare utilization, in 2017-18, for-profit formal private sector utilization was 59.1% whereas for NGOs and Trust hospitals it was 3.6%. Public healthcare utilization was higher in rural areas (38.7%) compared to urban areas (35.2%), male (39.2%) compared to females (35.7%), ST category (65.1%) compared to general category (24.1%), and rural poorest (46.2%) compared to urban richest (14.1%).

**Table 2:** Share of public and private providers for hospitalization in Kerala in 2014 and 2017-18, by background characteristics

	71st Round, 2014 (*n* = 3002)		75th Round, 2017-18 (*n* = 4986)			
	Pub(%)	Pvt. (%)	Pub (%)	Pvt. (%)	Trust/NGO (%)	Pvt. Total* (%)
**Total**	33.9	66.2	37.3	59.1	3.6	62.7
**Rural-urban divide**						
Rural	34.4	65.6	38.7	58.3	3.0	61.3
Urban	33.0	67.0	35.2	60.3	4.5	64.8
**Gender**						
Male	36.6	63.4	39.2	56.6	4.3	60.9
Female	31.8	68.2	35.7	61.3	3.0	64.3
**Social Group**						
ST	69.4	30.6	65.1	34.2	0.7	34.9
SC	55.7	44.3	59.3	39.5	1.2	40.7
OBC	34.7	65.3	40.1	56.6	3.3	59.9
GEN	23.0	77.0	24.1	71.0	4.9	75.9
**Economic Class**						
**Rural**						
Poorest	48.6	51.4	46.2	52.1	1.7	53.8
Poor	46.1	53.9	41.1	54.7	4.2	58.9
Middle	37.2	62.8	45.6	50.6	3.8	54.4
Rich	23.5	76.5	31.0	64.6	4.5	69.1
Richest	20.2	79.8	23.5	75.2	1.3	76.5
**Urban**						
Poorest	49.3	50.7	40.2	55.3	4.5	59.8
Poor	40.6	59.4	43.0	55.1	1.9	57
Middle	27.8	72.3	40.8	52.0	7.2	59.2
Rich	26.6	73.4	26.1	68.4	5.5	73.9
Richest	15.1	84.9	14.1	80.5	5.4	85.9

* Private total includes for profit private provider and trust/NGO

Source: Authors’ computation from unit records of NSSO (71st Round 2014 and 75th Round 2017-18)

#### Outpatient care

Public healthcare utilization in Kerala increased from 34.0%, in 2014, to 47.5% in 2017-18 (Table 3). Proportional increase for public healthcare utilization was higher in rural areas (42.7%) compared to urban (34.1%) areas. Public healthcare utilization for the outpatient care was higher for the ST category (67.6%) compared to general category (34.0%), and rural poorest quintile (61.3%) than to urban richest quintile (20.5%). Overall informal healthcare utilization in Kerala was 0.2% and it was highest in the urban richest (1.5%) population (Table 3).

**Table 3:** Share of public and private providers for out-patient care in Kerala in 2014 and 2017-18, by background characteristics

	71st Round, 2014 (*n* = 3385)		75th Round, 2017-18 (*n* = 6070)				
	Pub (%)	Pvt. (%)	Pub (%)	Pvt. (%)	Trust/NGO (%)	Informal (%)	Pvt. Total*(%)
**Total**	34.0	66.0	47.5	50.9	1.4	0.2	52.3
**Rural-urban divide**							
Rural	36.3	63.7	51.8	46.7	1.5	0.0	48.2
Urban	31.1	68.9	41.7	56.5	1.3	0.4	57.8
**Gender**							
Male	31.3	68.7	47.2	50.9	1.7	0.3	52.6
Female	36.0	64.0	47.7	50.8	1.3	0.1	52.1
**Social Group**							
ST	13.9	86.2	67.6	32.4	0.0	0.0	32.4
SC	57.7	42.3	66.1	32.6	1.3	0.0	33.9
OBC	33.3	66.7	53.1	45.3	1.2	0.4	46.5
GEN	28.2	71.9	34.0	64.1	1.9	0.0	66.0
**Economic Class**							
**Rural**							
Poorest	55.9	44.2	61.3	37.9	0.8	0.0	38.7
Poor	38.6	61.4	59.0	39.7	1.2	0.0	40.9
Middle	38.9	61.1	53.7	44.5	1.9	0.0	46.4
Rich	25.7	74.4	47.5	49.5	2.9	0.1	52.4
Richest	21.9	78.1	37.9	61.2	0.9	0.0	62.1
**Urban**							
Poorest	44.2	55.8	53.5	45.2	0.7	0.7	45.9
Poor	28.6	71.4	51.6	47.5	0.5	0.4	48.0
Middle	34.4	65.6	49.8	48.0	2.2	0.0	50.2
Rich	23.5	76.5	26.5	71.1	2.4	0.0	73.5
Richest	20.3	79.7	20.5	77.3	0.9	1.5	78.2

* Private total includes for profit private provider and trust/NGO

Source: Authors’ computation from unit records of NSSO (71st Round 2014 and 75th Round 2017-18)

### Financial protection

Financial protection was measured in terms of health insurance coverage, OOPE, CHE-10 and CHE-25.

#### Hospitalization

Health insurance coverage by PFHI dropped to 32.8%, in 2017-18, from 34.6% in 2014 (Additional File S1). PFHI coverage was higher in rural areas (36.8%) than urban areas (27.9%), female (33.6%) compared to male (32.0%), ST category (44.1%) than general (25.7%), and rural poorest (39.9%) compared to urban richest (15.3%).

Overall average OOPE per hospitalization in public sector, increased from Rs. 3153 in 2014 to Rs. 4373 in 2017-18, in Kerala (Table 4). Whereas in private sector, average OOPE per hospitalization increased from Rs. 22,974 to Rs. 26,363 in the same time period. Average OOPE per hospitalization under NGO and trust hospitals was Rs. 18,318. OOPE under private sector increases as we move from the marginalized and poor population groups to higher socioeconomic population groups. For example, average OOPE per hospitalization for the urban richest was Rs. 37,705 whereas for rural poorest it was Rs. 20,223. Under public sector average OOPE for the rural areas (Rs. 4543) was higher than the urban areas (Rs. 4104). However, average OOPE under public sector was higher in the richest quintiles compared to the poorest quintiles.

**Table 4:** Out-of-pocket expenditure (OOPE) for *hospitalization* in Kerala in 2014 and 2017-18 by background characteristics

	71st Round, 2014 (*n* = 3002)		75th Round, 2017-18 (*n* = 4986)			
	Pub (in INR)	Pvt. (in INR)	Pub(in INR)	Pvt. (for profit) (in INR)	Trust/NGO (in INR)	Private total*(in INR)
**Total**	**3153**	**22,974**	**4373**	**26,853**	**18,318**	**26,363**
**Rural-urban divide**						
Rural	3294	24,904	4543	24,980	15,632	24,528
Urban	2934	20,163	4104	29,464	20,863	28,865
**Gender**						
Male	3784	30,814	4868	30,583	22,172	29,993
Female	2595	17,384	3920	23,978	13,796	23,497
**Social Group**						
ST	4943	8708	3049	9972	5382	9881
SC	2118	16,010	3803	21,562	18,240	21,464
OBC	3041	24,743	4187	24,048	16,348	23,619
GEN	4372	21,116	5554	32,807	21,294	32,060
**Economic Class**						
**Rural**						
Poorest	1726	11,765	4363	20,454	13,204	20,223
Poor	2757	10,749	5319	20,809	12,232	20,198
Middle	1721	18,913	3131	23,930	28,843	24,275
Rich	5240	16,741	5823	26,098	11,411	25,142
Richest	8215	47,263	4662	33,852	9530	33,433
**Urban**						
Poorest	2875	18,694	3479	26,069	21,617	25,734
Poor	2653	20,075	4192	24,807	13,639	24,432
Middle	3222	12,225	4119	26,076	5745	23,594
Rich	2618	19,574	3831	35,725	14,917	34,181
Richest	4296	30,614	7736	36,288	58,855	37,705

* Private total includes for profit private provider and trust/NGO

Source: Authors’ computation from unit records of NSSO (71st Round 2014 and 75th Round 2017-18)

CHE-10 during public hospitalization in Kerala decreased from 16.6%, in 2014, to 16.0% in 2017-18; whereas for the private hospitalization it increased from 49.5 to 55.8% for the same time period. Similarly, CHE-25 for the private sector increased from 22.3 to 26.2% for the same time period (See Table 5). CHE-10 and CHE-25 was considerably higher in the lower socioeconomic population compared to upper socioeconomic population in Kerala. For example, CHE-10, during private hospitalization, for urban poorest quintile was 74.5% whereas for the urban richest quintile it was 50.8% (Table 5).

**Table 5:** CHE-10 and CHE-25 during hospitalization in Kerala in 2014 and 2017-18 by background characteristics

	(CHE-10)						(CHE-25)						
	71st Round, 2014 (*n* = 1869)		75th Round, 2017-18 (*n* = 3325)				71st Round, 2014 (*n* = 1869)		75th Round, 2017-18 (*n* = 3325)				
	Pub (%)	Pvt. (%)	Pub (%)	Pvt. (%)	Trust/NGO (%)	Pvt. Total*(%)	Pub(%)	Pvt. (%)	Pub (%)	Pvt. (%)	Trust/NGO (%)	Pvt. Total*(%)	
**Total**	16.6	49.5	16.0	56.9	36.7	55.8	6.8	22.3	6.8	26.6	19.7	26.2	
**Rural-urban divide**													
Rural	18.1	507	19.6	54.6	41.8	54.1	7.6	22.1	8.3	26.3	22.9	26.1	
Urban	14.4	48.0	11.2	60.0	32.3	58.1	5.7	22.6	4.8	27.0	17.0	26.3	
**Gender**													
Male	16.3	49.3	20.8	59.2	40.5	57.9	4.0	26.6	9.4	29.8	23.7	29.4	
Female	16.9	49.7	11.5	55.1	32.0	54.0	9.3	18.9	4.3	23.9	15.0	23.5	
**Social Group**													
ST	27.9	41.7	1.9	30.4	0.0	29.6	8.0	14.0	1.3	7.8	0.0	7.6	
SC	18.4	45.2	13.9	52.2	53.2	52.3	13.3	20.8	6.7	25.9	37.3	26.4	
OBC	14.9	51.6	15.9	56.7	31.9	55.2	4.4	24.0	6.9	25.6	18.2	25.2	
GEN	19.9	46.6	19.0	58.5	44.5	57.8	9.1	19.5	7.0	28.6	20.7	28.2	
**Economic Class**													
**Rural**													
Poorest	13.6	95.6	24.8	61.0	52.3	60.6	3.6	66.1	15.8	26.1	52.3	27.2	
Poor	3.4	65.8	31.0	75.9	0.0	74.1	0.0	30.4	11.7	51.1	0.0	49.9	
Middle	17.6	46.8	17.3	57.2	46.4	56.5	5.5	20.7	10.8	27.5	17.6	26.9	
Rich	13.3	46.0	23.1	57.9	84.2	58.4	0.2	11.7	10.4	26.4	84.2	27.5	
Richest	21.2	51.1	17.9	52.0	37.5	51.3	11.6	23.8	6.2	24.8	17.2	24.4	
**Urban**													
Poorest	32.6	70.7	15.6	76.8	36.9	74.5	12.3	56.0	8.4	43.0	29.5	42.3	
Poor	3.1	56.9	13.8	49.9	51.2	50.0	3.1	29.8	2.7	20.8	20.1	20.8	
Middle	12.2	40.7	9.5	64.5	12.7	58.5	5.4	19.5	4.9	30.8	1.8	27.4	
Rich	17.7	45.2	3.7	56.6	45.9	56.0	4.9	18.5	2.4	21.5	23.5	21.6	
Richest	20.5	50.3	8.5	51.1	47.2	50.8	5.3	19.6	4.7	17.6	31.2	18.6	

* Private total includes for profit private provider and trust/NGO

Source: Authors’ computation from unit records of NSSO (71st Round 2014 and 75th Round 2017-18)

Average OOPE under private sector without PFHI coverage was RS. 29,353, whereas with PFHI coverage under private sector it decreases to Rs. 21,597. Similarly, under public sector with PFHI OOPE was Rs. 4705, whereas without PFHI coverage it was Rs. 3601 (Table 6).

**Table 6:** Financial Protection under PFHI and type of service provider in Kerala in 2014 and 2017-18

	71st Round, 2014				75th Round, 2017-18							
	Private provider without any insurance	Private provider with PFHI	Public provider without any insurance	Public provider with PFHI	Private provider without any insurance	Private provider with PFHI	Public provider without any insurance	Public provider with PFHI	Trust Hospital without any insurance	Trust hospital with PFHI	Total Private provider without any insurance*	Total Private provider with PFHI
Mean OOPE per hospitalization (in INR)	26,253	13,199	3619	2178	29,353	21,597	4705	3601	28,354	7687	29,304	20,315
Median OOPE per hospitalization (in INR)	8950	6650	1450	720	14,850	10,250	1700	1700	10,870	3600	14,740	9200
% of hospitalization episodes with OOPE < 500 Rs.	2.33	6.4	23.7	40.0	0.1	2.1	24.3	23.6	1.8	15.8	0.2	3.4
% of hospitalization episodes with OOPE < 1000 Rs.	4.5	10.0	40.9	55.1	0.8	5.1	38.6	3.7	3.7	16.8	0.9	6.2
% of hospitalization episodes with OOPE < 3000 Rs.	17.1	25.8	74.0	82.3	6.9	15.7	62.4	68.0	16.3	28.4	7.4	16.9
% of hospitalization episodes with OOPE < 5000 Rs.	29.9	39.0	82.5	90.8	15.1	28.1	74.9	80.9	27.0	63.0	15.7	31.3
Incidence of CHE-10 (%)	49.3	49.7	14.6	15.8	27.8	24.5	17.8	13.5	50.2	21.6	59.3	50.0
Incidence of CHE-25 (%)	22.6	20.8	3.0	7.5	59.8	52.7	8.2	5.3	29.2	10.8	27.6	23.3

* Private total includes for profit private provider and trust/NGO

Source: Authors’ computation from unit records of NSSO (71st Round 2014 and 75th Round 2017-18)

#### Outpatient care

Average OOPE under public sector increased from Rs. 221 in 2014, to Rs. 239 in 2017-18 (Table 7). On the other hand, average OOPE under private sector increased from Rs. 564 to Rs. 795 for the same time period. Average OOPE under trust and NGOs was Rs. 800. However, average OOPE under informal provider was Rs. 11,439, and it was significantly higher in urban areas (Rs. 12,475) compared to the rural areas (Rs. 507). Average OOPE under public facility, for outpatient care, for the rural poorest population was Rs. 180 whereas it was Rs. 498 for the urban richest quintile in 2017-18 (Table 7).

**Table 7:** OOPE for out-patient care under public and private provider in Kerala in 2014 and 2017-18, by background characteristics

	71st Round, 2014 (*n* = 3385)		75th Round, 2017-18 (*n* = 6070)				
	Pub (in INR)	Pvt. (in INR)	Pub (in INR)	Pvt. (in INR)	Trust/NGO (in INR)	Informal (in INR)	Pvt total* (in INR)
**Total**	**221**	**564**	**239**	**752**	**800**	**11,439**	**795**
**Rural-urban divide**							
Rural	201	545	202	794	944	507	798
Urban	250	587	301	706	579	12,475	791
**Gender**							
Male	265	573	261	813	1135	18,425	917
Female	189	557	223	707	474	1065	701
**Social Group**							
ST	56	782	205	306	-	-	306
SC	288	401	178	619	270	-	606
OBC	192	551	251	672	683	11,438	752
GEN	259	623	232	864	1001		868
**Economic Class**							
**Rural**							
Poorest	168	338	180	712	408	218	705
Poor	129	396	181	979	364	165	959
Middle	147	577	241	646	605	-	645
Rich	372	588	178	740	1205	810	766
Richest	396	727	250	876	1877	-	891
**Urban**							
Poorest	241	457	260	655	243	1285	658
Poor	189	451	250	611	574	510	610
Middle	257	455	416	983	508	-	962
Rich	293	856	235	707	678	-	706
Richest	310	725	498	635	760	27,000	1118

* Private total includes for profit private provider, trust/NGO and informal provider

Source: Authors’ computation from unit records of NSSO (71st Round 2014 and 75th Round 2017-18)

## Discussion

The findings from the study have shown a decline in hospitalization rate and PAP as compared to 2014 figures, which was quite unusual for Kerala’s scenario, but similar trend was observed at all-India level as well [21]. The plausible reason could be the impact of de-monetization implemented in end of 2016, which has reduced consumption of healthcare in the country as well as State [25, 26]. Women were having high PAP as compared to men but the hospitalization rate was low among women. For instance, our study shows that, average OOPE for hospitalization was significantly lower for the women member of the household than male member, which is an indication of gender-based discrimination by the household in allocation of resources for the healthcare needs even though women were having higher PAP. Even after excluding child birth from average OOPE, calculation pattern remains the same [22]. Previous studies have also indicated similar findings [14, 27–29]. Similarly, ST population and the poorest economic category has shown lowest PAP as well as hospitalization rate (except rural poor with lowest hospitalization rate than rural poorest). The weaker sections in society have high levels of latent disease due to either a lack of appropriate healthcare seeking and awareness, competing demands of work and care and different barriers that together contribute to care foregone, resulting in low hospitalizations rate. The findings reiterate the fact that gender, caste and class structure are important social determinant of health in Kerala. Similar findings were also reported by previous studies [30, 31]. This leads to a situation where though Kerala has one of the highest levels of healthcare utilization, there is considerable unmet needs in the poorer economic quintiles and more marginalized communities. There is currently a pressing need from the vibrant scientific community of Kerala to improve awareness and address the social determinants of health in the State to tackle these existing health inequalities [32].

Income and wealth inequity is not low in this state, and Kerala’s Gini Co-efficient shows higher inequity [33]. There are very few large-scale studies that have evaluated the direct linkages between health outcomes and social determinants in Kerala. One notable earlier study by the Kerala Sastra Sahitya Parishad (KSSP) has shown that the mortality and morbidity patterns was having an inverse relationship with socio-economic status [34]. There are studies also showing caste affiliation as an independent social determinant of health outcomes in low socio-economic groups in Kerala [32, 34].

Infectious diseases, followed by cardiovascular conditions and injuries were the major reasons for hospitalization. For outpatient care, cardiovascular conditions and diabetes were the major causes. This is in tune with the epidemiological profile of the State [2, 35]. Kerala has now completed an epidemiological and demographic transition. Kerala faces a high burden of preventable, premature mortality due to non-communicable diseases including mental health and due to injuries [36, 37], and has also been facing repeated outbreaks of either altogether new infectious diseases or sporadic re-emergence of old ones [38, 39]. The share of hospitalization episodes treated under public sector was highest for blood diseases followed by Cancers. Blood diseases and cancer are usually associated with high OOPE and people prefer to go to public sector; there is also a strong public sector presence for Cancer Care (for example, the Regional Cancer Centre (RCC), Thiruvananthapuram). Similar findings were also reported by other studies [40–42].

Due to better health literacy and health seeking behaviour, there is relatively higher utilization of Out-Patient services in Kerala, but segmented. The utilization rate which is used as a proxy indicator for access to healthcare in the study reveals that, there is an overall increase in public healthcare utilization for both IP and OP care; with the percentage increase being significantly higher for OP care (13.5% increase). The public healthcare utilization for OP care was ranging between 28 - 37% for rural and 22 -33% for urban during last four NSS rounds for health from 1986 - 87 to 2014-15. The 75th round in 2017-18 was the first time it crossed 50% for rural and 40% for urban. This would be consistent with the expectation that as more services of better quality become available in public sector, sections of middle class would shift back to public sector utilization because of the financial protection it provides. This is also way higher than the overall all-India utilization of public facilities for OP care (30.2%) [4, 21]. For IP care, even though there is an increase in public healthcare utilization, the percentage change in rural and urban is marginal and lesser than the all-India Fig. (42%). Reasons behind these patterns could be further explored through additional studies.

In the 75th NSS round, the utilization of public sector was more not only in SC and ST category, but also in OBC sub-group, and in the poorest three quintiles of rural areas and urban areas. The disaggregated results on OP utilization shows that, there is an increase in public healthcare utilization among all social and class groups from 2014 to 2018. Here, the ST group has shown almost five times increase in public healthcare utilization, which is remarkable from the equity point of view. The OBC social group and the mid economic quintiles (Rural & Urban) have also shown a considerable increasing trend in public healthcare utilization. One of the possible reasons behind this could be higher public investment in the healthcare from the state [7, 43, 44]. Public investment in the healthcare has always been priority area for the state since its formation [45]. Secondly, it could be also higher resilience of the public healthcare facilities during the time of demonetization in the state where private sector refused to provide care whereas public sector was more accessible to all sections of society [25, 46]. All the various categories (social and economic) in Kerala have shown an increased share in public sector OP care utilization in comparison with all-India disaggregated figures for same categories [21]. Overall, the rural population utilizes public sector more than private sector, and the reverse is true for urban areas. These are early indications that the UHC reforms with focus on PHC strengthening in the State is yielding better results in terms of increasing out-patient health service utilization across all population sub-groups.

For hospitalization, there is a decline in public healthcare utilization for both ST and SC social group in 2018 as compared to 2014; similar downward trend is noted among rural poorest & poor as well as among urban poorest category. A very marginal decline in public healthcare utilization is noted among urban rich and richest as well. However, all the various social groups, except the General category are showing better public service IP utilization than all-India figures. But, under various economic quintiles, all- India figures are better than Kerala for public sector hospitalization [21]. The reasons for this have to be further studied. In Kerala’s context, it’s not just socio-economic status but the issues like the access to more high technology services, perception of people regarding service quality, and capacity to access appropriate health care facility also determine public healthcare utilization [1, 3, 7, 44].

The overall OOPE for hospitalization as well as OP in the public sector is low as compared to private sector. The overall OOPE for both public as well as private is also lesser as compared to all-India figures [4, 21]. However the gap between public versus private has got widened as we compare 2014 to 2018. OOPE under public and private sector increases as we move from the ST to General category for IP care. For OP care, SC is having low OOPE as compared to ST and General category is having lower than OBC for public sector. The difference in OOPE between each social group tends to be minimal for OP care which points towards an efficient PHC system which is offering financial protection. Under the various economic categories under public sector, rural middle class and urban poorest were shown to be having lowest OOPE for IP care and rural rich and urban rich were shown to be having lowest OOPE for OP care.

The overall CHE-10 during public hospitalization has shown a marginal decline but remained static for CHE-25. However, CHE-10 and CHE-25 has shown an increasing trend for private sector hospitalization which is a worrying trend. Across all social groups, there seems to be a declining trend at CHE-10 and CHE-25 for public sector except OBC, where there is an increase. The findings shows that only 1–2% of ST category has faced CHE (both CHE-10 & 25) under public sector, which is way lower than the all-India figures of 14% (CHE-10) and 4% (CHE-25). Under the economic class category, both rural and urban poorest and rural poor and urban poorest faced maximum CHE-25 under public and private sector respectively.

Both the NSS rounds (2014 &2018) show that coverage with PFHI is 34.6% and 32.8% of the population in Kerala. This is generally a modest figure than can be expected from government data- but it is still a higher coverage as compared to most other states [4]. However, insurance coverage does not seem to lead to cashless services (financial protection) - and the costliest care in public sector without insurance is still cheaper than the lowest rates with insurance in the private sector. The mean OOPE difference is less than Rs 8000 under private sector with and without PFHI coverage. There is also no marked difference in incidence of CHE-10 and 25 as well for both categories. But, it has an impact on percentage of hospitalization episodes with OOPE (less than 1000–5000 Rs range). Similarly, under public sector there is no marked variation of mean OOPE per hospitalization for public provider with and without PFHI. A recent household survey in Kerala by Sharma et al. also evidenced that there is only marginal difference between median OOPE for hospitalization among insured as compared to non-insured [14]. This points to the fact that, the insurance as a financing tool to achieve UHC, especially with the engagement of private sector is not likely to contribute to adequate financial protection and sustainable health outcomes as far as Kerala is concerned, especially from the equity lens.

### Lessons from Kerala through a socio-political lens

Kerala has always had a strong commitment to both public education and healthcare- which are the hallmark of ‘Kerala model of development’. It is an established fact that different nations as well as different states would perform differently with regards to their level of health. The health outcomes of a nation/ state are mostly dependent on social, economic, historic, and political determinants than health systems performance itself [1, 47]. Kerala ranks top in the country among many of the social determinants like literacy (especially female literacy), access to safe drinking water, sanitation. The early introduction of land reform movements after Independence, creation of a robust public distribution system which made essential food items available at subsidized costs had influence on the relatively lower levels of poverty and malnutrition in the State- though there are significant levels. The history and evolution of PHC in Kerala was always rooted in public service strengthening along with strong community involvement even before the decentralization act of 1996 [48].

Kerala is one among the very few States where it has been governed by a coalition of left parties- for about half of the years since Independence and the other half by a centrist coalition of parties, led by the Congress – both pursuing what could be called a liberal economic policy with a commitment to a welfare state. The State has the highest degree of decentralization among all States- wherein almost 42% of the state budget is allocated through elected local self-government bodies, which are powerful. Substantial parts of public services are placed under the supervision, even ownership of local self-governments with strong community participation. The major part of primary health care is devolved to the local elected self-governments (LSG) (called panchayats) in the State. A recent study has shown that the decentralised governance structures in Kerala as part of LSGs enabled re-engineering of PHCs as part of Aardram by mobilisation of financial resources, provision of human resources, infrastructure modification, and enhanced community participation at various levels [49].

There has always been a high level of debate among civil society and media on public policy, thereby having a higher level of citizen engagement in public policy making. There is also a progressive pro-active public health academic community, which has had a focus on health rights and influence over the Directorates. This enabled the state government to be able to take greater state level innovative initiatives, which are context specific and the state does not have to rely only on the technical design of central programs or on external donor/funding agencies. All of these have major implications for the design and delivery of healthcare systems in Kerala of which Aardram is a good example.

Kerala currently faces a high burden of preventable, premature mortality due to non-communicable diseases including mental health and due to injuries. Though Kerala built a robust healthcare system, its primary care system was utilised more for delivering a selective package of services. The increasing burden of chronic illness was left to the care of private sector. The Study also revealed that the proportion of people going to public sector for outpatient care is more in Kerala than the all-India average- both in 2014 and in 2017. Whereas when it comes to in-patient care, the proportion of population going to public sector in Kerala is less than all India average. The State has a long history of investments in public services including in healthcare. But, in the course of time, the public health systems were not re-designed or expanded as required to address the new epidemiological and demographic situation in the State. The dependance on various insurance mechanisms for increasing access to secondary care as compared to expanding and strengthening public hospital capacity resulted in an acceleration of growth of an unregulated private health sector, where health outcomes are uncertain- but there is a high incidence of financial hardship and impoverishment due to healthcare expenditure. The UHC reforms with currently a focus on PHC strengthening were initiated in the State as part of addressing these challenges in healthcare delivery [49].

The initial focus of Aardram was creating people friendly quality healthcare delivery systems in the state from primary health centers to medical colleges and treating every patient with dignity based on their needs. This was indirectly aiming to address perceived quality as related to patient experience in seeking out-patient care in public facilities. From comfortable waiting areas with good seating arrangements to disabled-friendly toilets, there were many infrastructure elements which contributed to enhancing patient experience. The re-engineered Primary Health Centres, now known as the Family Health Centres (FHC) had an expansion in human resources and larger assured set of PHC services as opposite to selective PHC [50]. In parallel to the FHCs, the state has also initiated a number of public health programs to address the new range of healthcare priorities including addressing social determinants. There were also efforts to strengthen the secondary and tertiary care institutions under Aardram to ensure continuum of care, but clearly this has not yet gone far enough.

The present study has thrown light into many aspects on Kerala’s journey towards UHC through an equity lens in that it shows changes across different socio-economic background characteristics. However, there are limitations of this study. First, many of the components of the Aardram program were at the nascent stage during the data collection period of NSS 75th rounds. The implementation of FHC’s started in 2017-18, with 170 institutions in first phase, 504 (2018-19) in phase 2 and 220 (2019-20) in phase 3 [51]. But currently the scale of implementation is not completed and hence the monitoring of outputs and outcomes may be possible in the next round of NSSO survey. Second, NSS is a self-reporting survey and their might be recall bias related with reporting of healthcare needs which has been pointed out in other studies [52].

Finally, implementation of the Aardram Mission is large enough for proof of the concept, but not large enough for a population wide impact. That may require creating additional public hospital capacity. Further, to ensure sustainability, there is currently an urgent need for good internal advocacy and community mobilization as well as financial investment so that the scaling up of this approach covers the entire state. As Aardram mission proceeds, demands raised by the community and local self-government to expand the scheme to all facilities is a push factor to the Government for its rapid scaling up. This sense of demand driven care, public ownership and trust can drive the program to greater heights.

## Conclusions

The major findings from the study, in terms of increased access / utilization of public facilities for OP care, reduced OOPE and equitable service delivery (through various disaggregated analysis) are very early signs of positive impacts and is indicative of the enhanced trust of the public healthcare delivery system in Kerala, post the launch of Universal Health Coverage reforms in the State. There are many outliers also, as evidenced by the study which needs further exploration. The study also underlines the lacunae and inadequacies of UHC reforms linked to publicly financed health insurance. We note that the level of financial protection provided by public sector to a non-insured person is more than the level of financial protection provided by insurance in the private sector.

Kerala, with a strong history of public provisioning and PHC always had a considerable focus on ‘social development’ throughout history. Kerala has to pay attention to expand and strengthen the services under PHC -not restricted to curative and ensuring continuum of care- both forward and backward. This requires above all a much greater effort at upgrading the health sub-centers in the State, to the level of functionality of the PHCs as strengthened by Aardram. The lack of attention to health sub-centers was a weak point during all the health systems strengthening efforts, especially from the point of view of “health promotion” to address several diverse emerging and existing health systems challenges in the State. It also has to expand the range and quality of services available at the public hospital and facilitate access to the same.

### Electronic supplementary material

Below is the link to the electronic supplementary material.

Supplementary Material 1

Supplementary Material 2

Supplementary Material 3

## Data Availability

This survey was conducted by the National Sample Survey Office, Ministry of Statistics and Program Implementation, Government of India and anonymized data was provided in the public domain for the research purposes.

## Abbreviations

AMPCE: Annual monthly per capita expenditure
CHE: Catastrophic health expenditure
CHIS: Comprehensive Health Insurance Scheme
FHC: Family Health Centres
INR: Indian Rupee
IP: In-Patient
KASP: Karunya Arogya Suraksha Padhathi
LSG: Local self-governments
NGO: Non-Governmental Organization
NSS: National Sample Survey
OBC: Other Backwards Classes
OOPE: Out-of-pocket expenditure
OP: Out-Patient
PFHI: Publically Funded Health Insurance
PMJAY: Pradhan Mantri Jan Arogya Yojana
PHC: Primary Healthcare
PAP: Proportion of ailing population
RSBY: Rashtriya Swasthya Bima Yojana
SC: Scheduled Castes
ST: Schedule Tribes
SHA: State Health Agency
UHC: Universal Health Coverage
UMPCE: Usual monthly per capita consumer expenditure
UAPCE: Usual annual per capita consumer expenditure
