# Supplementary material for: Kerala’s progress towards universal health coverage: the road travelled and beyond
Source: Int J Equity Health. 2024 Aug 5;23:152. doi: 10.1186/s12939-024-02231-2 (PMC11302021; doi:10.1186/s12939-024-02231-2)
Supplement: Supplementary file 1 — Supplementary Material 1 [file 12939_2024_2231_MOESM1_ESM.docx]

**Additional Table 1: Sample background characteristics, hospitalization rate, proportion of ailing population (PAP), health insurance coverage, catastrophic health expenditure at 10% (CHE-10) threshold in Kerala**

|  | Sample characteristics (N) | | Hospitalization rate (per 100 population) | | PAP ( per 100 population) | | PFHIs coverage (per 100 population) | | CHE-10 | |
| --- | --- | --- | --- | --- | --- | --- | --- | --- | --- | --- |
|  | 71^st^ (N) | 75^th^ (N) | 71^st^ (%) | 75^th^ (%) | 71^st^ (%) | 75^th^ (%) | 71^st^ (%) | 75^th^ (%) | 71^st^ (%) | 75^th^ (%) |
| **Total** | 11229 | 19801 | 12.1 | 9.9 | 30.8 | 24.5 | 34.6 | 32.8 | 37.8 | 41.2 |
| **Rural-urban divide** |  |  |  |  |  |  |  |  |  |  |
| Rural | 5484 | 10682 | 13.1 | 10.7 | 31.0 | 25.4 | 38.8 | 36.8 | 38.6 | 41.2 |
| Urban | 5745 | 9119 | 10.7 | 8.9 | 30.6 | 23.3 | 29.5 | 27.9 | 36.7 | 41.1 |
| **Gender** |  |  |  |  |  |  |  |  |  |  |
| Male | 5215 | 9358 | 11.4 | 10.5 | 29.3 | 22.6 | 35.4 | 32.0 | 37.2 | 43.8 |
| Female | 6014 | 10457 | 11.4 | 9.3 | 32.3 | 26.3 | 33.9 | 33.6 | 38.3 | 38.9 |
| **Social Group** |  |  |  |  |  |  |  |  |  |  |
| ST | 131 | 271 | 4.2 | 7.1 | 23.3 | 12.4 | 27.4 | 44.1 | 31.9 | 11.0 |
| SC | 1076 | 1484 | 11.9 | 10.3 | 28.4 | 21.2 | 60.1 | 40.1 | 29.1 | 30.4 |
| OBC | 7206 | 12631 | 11.0 | 10.0 | 30.3 | 24.1 | 33.6 | 35.1 | 38.2 | 39.7 |
| GEN | 2816 | 5492 | 12.7 | 9.6 | 33.5 | 26.6 | 26.7 | 25.7 | 40.3 | 48.2 |
| **Economic Class** |  |  |  |  |  |  |  |  |  |  |
| **Rural** |  |  |  |  |  |  |  |  |  |  |
| Poorest | 1349 | 3710 | 9.8 | 9.9 | 25.8 | 19.6 | 51.3 | 39.9 | 33.1 | 41.1 |
| Poor | 1190 | 2164 | 9.6 | 9.5 | 30.1 | 22.9 | 37.0 | 41.6 | 40.2 | 58.0 |
| Middle | 1310 | 1873 | 13.6 | 10.8 | 32.2 | 27.0 | 36.5 | 39.1 | 33.8 | 38.0 |
| Rich | 815 | 1797 | 14.4 | 10.8 | 34.3 | 27.6 | 30.7 | 33.6 | 30.1 | 45.3 |
| Richest | 820 | 1147 | 21.1 | 14.0 | 35.7 | 39.8 | 33.0 | 22.1 | 41.5 | 39.7 |
| **Urban** |  |  |  |  |  |  |  |  |  |  |
| Poorest | 1656 | 2863 | 7.8 | 8.0 | 29.3 | 20.6 | 36.6 | 31.1 | 48.6 | 50.4 |
| Poor | 137 | 2525 | 11.1 | 8.8 | 28.2 | 20.6 | 26.2 | 36.2 | 32.9 | 34.4 |
| Middle | 1060 | 1295 | 11.0 | 9.5 | 30.0 | 28.1 | 36.1 | 23.9 | 29.7 | 37.8 |
| Rich | 875 | 1446 | 12.3 | 8.7 | 30.5 | 26.1 | 23.8 | 21.8 | 38.0 | 40.8 |
| Richest | 779 | 995 | 13.4 | 10.9 | 37.5 | 25.7 | 18.1 | 15.3 | 46.2 | 44.5 |
| **Occupation** |  |  |  |  |  |  |  |  |  |  |
| Self-employed | 3693 | 6266 | 10.2 | 9.7 | 32.3 | 24.6 | 28.1 | 33.3 | 40.8 | 40.2 |
| Regular wages | 3306 | 4546 | 12.3 | 8.6 | 26.8 | 21.1 | 36.1 | 22.4 | 32.5 | 41.2 |
| Casual labour | 2783 | 6229 | 11.9 | 10.2 | 30.3 | 22.8 | 49.8 | 43.1 | 31.7 | 33.4 |
| Other | 1447 | 2774 | 12.0 | 11.3 | 36.2 | 32.7 | 22.0 | 26.0 | 47.4 | 53.6 |
| **Education** |  |  |  |  |  |  |  |  |  |  |
| Not literate | 1940 | 2810 | 16.3 | 15.7 | 37.8 | 29.6 | 26.1 | 22.5 | 27.5 | 30.2 |
| Up to primary level | 3118 | 5321 | 13.8 | 12.1 | 36.3 | 31.6 | 40.8 | 35.0 | 39.8 | 37.1 |
| Up to secondary level | 3695 | 6351 | 9.8 | 9.3 | 29.6 | 24.8 | 38.7 | 39.3 | 35.4 | 39.6 |
| Above secondary | 2476 | 5333 | 7.9 | 6.4 | 21.7 | 15.6 | 24.6 | 24.4 | 42.7 | 49.5 |
| **Provider for hospitalization** |  |  |  |  |  |  |  |  |  |  |
| Public | 981 | 1678 | NA | NA | NA | NA | NA | NA | 16.6 | 16.0 |
| Formal private | NA | 3122 | NA | NA | NA | NA | NA | NA | NA | 56.9 |
| Trust/NGO | NA | 186 | NA | NA | NA | NA | NA | NA | NA | 36.7 |
| Private total | 2021 | 3308 | NA | NA | NA | NA | NA | NA | 49.5 | 55.8 |
| **Provider for outpatient care** |  |  |  |  |  |  |  |  |  |  |
| Public | 1363 | 2544 | NA | NA | NA | NA | NA | NA | NA | NA |
| Formal private | NA | 3115 | NA | NA | NA | NA | NA | NA | NA | NA |
| Trust/NGO | NA | 110 | NA | NA | NA | NA | NA | NA | NA | NA |
| Informal | NA | 9 | NA | NA | NA | NA | NA | NA | NA | NA |
| Private total | 4166 | 3234 | NA | NA | NA | NA | NA | NA | NA | NA |
