# Supplementary material for: Kerala’s progress towards universal health coverage: the road travelled and beyond
Source: Int J Equity Health. 2024 Aug 5;23:152. doi: 10.1186/s12939-024-02231-2 (PMC11302021; doi:10.1186/s12939-024-02231-2)
Supplement: Supplementary file 2 — Supplementary Material 2 [file 12939_2024_2231_MOESM2_ESM.docx]

**Additional Table 2: Disease burden and public healthcare utilization during hospitalization and out-patient care in Kerala**

|  | **Hospitalization (N=4,986)** | | **Out-patient care (N=6,070)** | |
| --- | --- | --- | --- | --- |
|  | **Disease-specific causes of hospitalization (%)** | **Share of hospitalization episodes treated under public sector (%)** | **Disease- specific cause for seeking out-patient care (%)** | **Share of out-patient care treated under public sector (%)** |
| Infection | 28.5 | 39.9 | 12.8 | 50.7 |
| Cancers | 3.1 | 54.5 | 0.5 | 69.8 |
| Blood diseases | 2.1 | 79.9 | 0.5 | 50.7 |
| Endocrine, metabolic, nutritional  (Includes diabetes) | 4.1 | 35.9 | 20.4 | 49.7 |
| Psychiatric and Neurological | 5.6 | 36.7 | 5.1 | 42.1 |
| Genito-urinary | 6.0 | 24.0 | 1.2 | 44.5 |
| Eye and Ear | 2.4 | 36.8 | 2.2 | 31.0 |
| Cardio-vascular (includes hypertension) | 12.9 | 38.9 | 27.1 | 51.1 |
| Respiratory | 6.5 | 50.5 | 8.8 | 48.9 |
| Gastro-Intestinal | 6.0 | 33.3 | 1.7 | 35.9 |
| Skin | 0.9 | 22.3 | 1.3 | 23.6 |
| Musculo-skeletal | 6.1 | 31.9 | 9.8 | 47.9 |
| Injuries | 9.4 | 32.8 | 0.9 | 26.6 |
| Others | 2.8 | 28.9 | 2.4 | 30.2 |
| Total | 100 |  | 100 |  |

Source: Authors’ computation from unit records of NSSO 75^th^ Round 2017-18
