# Supplementary material for: Kerala’s progress towards universal health coverage: the road travelled and beyond
Source: Int J Equity Health. 2024 Aug 5;23:152. doi: 10.1186/s12939-024-02231-2 (PMC11302021; doi:10.1186/s12939-024-02231-2)
Supplement: Supplementary file 3 — Supplementary Material 3 [file 12939_2024_2231_MOESM3_ESM.pdf]

സാർവ്വലൗകിക ആരോഗ്യ പരിരക്ഷയിലേക്കുള്ള കേരളത്തിന്റെ പുരോഗതി : ഒരു അവലോകനം

സംഗ്രഹം

പശ്ചാത്തലം

കഴിഞ്ഞ ദശകത്തിൽ തന്നെ ആരോഗ്യ പരിരക്ഷ സാർവ്വലൗകികമാക്കുന്നതിനു (Universal Health Coverage-UHC) വേണ്ടി ഒട്ടേറെ പരിഷ്ക്കാരങ്ങൾ കേരളത്തിൽ നടപ്പിലാക്കുക ഉണ്ടായി. ഈ രംഗത്തു എടുത്തു പറയേണ്ടത് 2017ൽ തുടങ്ങിയ സംസ്ഥാന തലത്തിലുള്ള ആർദ്രം പദ്ധതിയിലെ പ്രവർത്തനങ്ങളാണ്. ഈ പദ്ധതിയുടെ ഉദ്ദേശ ലക്ഷ്യങ്ങൾ, വ്യാപ്തി, സമർപ്പണ ക്ഷമത മുതലായ ഘടകങ്ങൾ കേന്ദ്രികരിച്ചതു പ്രാദേശിക തലത്തിലുള്ള പ്രാഥമിക ആരോഗ്യകേന്ദ്രങ്ങളെ ശക്തിപ്പെടുത്തുക എന്നതിലായിരുന്നു. കഴിഞ്ഞ ദശകത്തിൽ കേരളത്തിൽ നടപ്പിലാക്കിയ പ്രധാന UHC പരിഷ്ക്കാരങ്ങളുടെ പശ്ചാത്തലത്തിൽ ആരോഗ്യ പരിരക്ഷമാർഗങ്ങൾ സാമാന്യ ജനങ്ങൾക്ക് എത്ര മാത്രം പ്രാപ്യമാണെന്നും, അതു മൂലമുള്ള സാമ്പത്തിക സുരക്ഷിതത്വവും ഈ പഠനത്തിലൂടെ വിശകലനം ചെയ്യുന്നു. ഒരു രാഷ്ട്രീയ അധിഷ്ഠിത സാമ്പത്തിക വ്യവസ്ഥിതിയിൽ (Political economy approach) നിന്ന് കൊണ്ട് സാർവലൗകിക ആരോഗ്യ പരിരക്ഷയിലും മറ്റു ആരോഗ്യരക്ഷ സംവിധാനങ്ങളിലും കേരളം സൃഷ്ടിച്ച മാറ്റങ്ങളെ എടുത്തു പറയുവാനും ഈ പഠനം വഴി ശ്രമിക്കുന്നുണ്ട്.

പഠന രീതി

2017-18 ലെ 75- ആം ദേശീയ സാമ്പിൾ സർവേയുടെ കേരള സംസ്ഥാന സാമ്പിളിൽ നിന്നുള്ള ഡാറ്റ ഈ പഠനത്തിനായി ഉപയോഗിച്ചിരിക്കുന്നു.

സാമാന്യ ജനങ്ങൾക്ക് ആരോഗ്യ പരിരക്ഷമാർഗങ്ങൾ എത്രമാത്രം പ്രാപ്യമാണെന്നും, അത് മൂലമുള്ള സാമ്പത്തിക പരിരക്ഷ എത്ര മാത്രം മെച്ചപ്പെട്ടു എന്ന് അളക്കുന്നതിനായി 2014 ലെ 71- ആം സാമ്പിൾ സർവേയുടെ ഫലങ്ങളുമായി ഉള്ള താരതമ്യപഠനവും നടത്തിയിരിക്കുന്നു. ലോജിസ്റ്റിക് റിഗ്രഷൻ (logistic regression) എന്ന സ്റ്റാറ്റിസ്റ്റിക്കൽ മാർഗ്ഗവും ഉപയോഗിച്ചു. ഈ കണ്ടെത്തലുകൾ ഒരു

രാഷ്ട്രീയ അധിഷ്ഠിത സാമ്പത്തിക വ്യവസ്ഥിതിയിലൂടെ വീണ്ടും വിശകലനം ചെയ്യിരിക്കുന്നു.

കണ്ടെത്തലുകൾ

പൊതുജനാരോഗ്യമേഖലയിൽ 2014 ൽ 34% മാത്രം ഉണ്ടായിരുന്ന ഔട്ട്പേഷ്യൻ്റ് കെയറിനുള്ള (OP care) ചികിത്സാ വിഹിതം 2017-18 കാലയളവിൽ 47.5% എന്ന തോതിലേക്കു വർദ്ധിച്ചു. സംസ്ഥാനത്തെ സാമൂഹികവും സാമ്പത്തികവുമായി താഴെക്കിടയിൽ ഉള്ളവർക്ക് പൊതുജനാരോഗ്യ കേന്ദ്രങ്ങളിൽ OP ചികിത്സാ സൗകര്യം ഗണ്യമായി വർദ്ധിച്ചു. കിടത്തി ചികിത്സക്കുള്ള (hospitalization) പൊതുമേഖലയുടെ വിഹിതം 2014 ലെ 33.9% ൽ നിന്നും, 2017-18 ൽ 37.3% ആയി ഉയർന്നതായി ഈ പഠനം കണ്ടെത്തി. എന്നിരുന്നാലും OP പരിചരണത്തിൽ കാണിക്കുന്ന വർദ്ധനവിൻറെ തോതിൽ അല്ല hospitalization ഇൽ ഉണ്ടായിട്ടുള്ള വർദ്ധനവ്. OP care, hospitalization, എന്നിവയ്ക്ക് പൊതുജനാരോഗ്യകേന്ദ്രങ്ങളെ അപേക്ഷിച്ചു സ്വകാര്യ ആശുപത്രികളിൽ രോഗി നേരിട്ട് ചിലവഴിച്ച തുക വളരെ അധികം വർദ്ധിച്ചതായും ഈ പഠനം കണ്ടെത്തി.

ഉപസംഹാരം

സംസ്ഥാനത്തു സാർവലൗകിക ആരോഗ്യരക്ഷാ പരിഷ്കാരങ്ങൾ (UHC) ആരംഭിച്ചതിനു ശേഷം OP care, hospitalization എന്നിവക്ക് പൊതുജനാരോഗ്യ കേന്ദ്രങ്ങളിൽ വന്നിട്ടുള്ള മൊത്തത്തിലുള്ള വർദ്ധനവ്, കേരളത്തിലെ പൊതുജനാരോഗ്യ സംവിധാനത്തിൽ ജനങ്ങൾക്കിടയിലുള്ള വർദ്ധിച്ച വിശ്വാസത്തെ സൂചിപ്പിക്കുന്നു. ഇൻഷുറൻസ് ബന്ധിത ആരോഗ്യ പരിരക്ഷാ സംവിധാനം കേരളത്തിന് UHC- ലേക്ക് കൂടുതൽ മുന്നേറുന്നതിനു അപര്യാപ്തമാണെന്ന് ഈ പഠനം വിലയിരുത്തുന്നു. ആരോഗ്യമേഖലയിൽ പൊതു സമ്പത്തിന്റെ വിനിയോഗം വിജയകരമായി നടപ്പിലാക്കിയ ദീർഘമായ ചരിത്രമുള്ള കേരളം, സാർവലൗകിക ആരോഗ്യപരിരക്ഷ (UHC) എന്ന ലക്ഷ്യം കൈവരിക്കാനായി ആർദ്രം മിഷൻ ന്റെ പ്രവർത്തിനങ്ങളിലൂടെ പ്രാഥമിക ആരോഗ്യ കേന്ദ്രങ്ങളെ ശക്തിപ്പെടുത്തുന്നതിൽ ശ്രദ്ധ കേന്ദ്രീകരിക്കണം എന്നും ഈ പഠനം വിലയിരുത്തുന്നു.
